# *Lavandula angustifolia* Effects on Rat Models of Alzheimer’s Disease Through the Investigation of Serum Metabolic Features Using NMR Metabolomics

**Published:** 2018

**Authors:** Afsaneh Arefi Oskouie, Reyhaneh Farrokhi Yekta, Mostafa Rezaei Tavirani, Masoud Soheili Kashani, Fatemeh Goshadrou

**Affiliations:** 1. Department of Basic Sciences, Faculty of Paramedical Sciences, Shahid Beheshti University of Medical Sciences, Tehran, Iran; 2. Proteomics Research Center, Faculty of Paramedical Sciences, Shahid Beheshti University of Medical Sciences, Tehran, Iran

**Keywords:** Alzheimer disease, Lavandula, Metabolomics, Serum

## Abstract

**Background:**

Alzheimer’s Disease (AD) is the most prevalent cause of memory impairment in the elderly population, but the diagnosis and treatment of the disease is still challenging. Lavender aqueous extract has recently been shown to have the potential in clearing Amyloid-beta plaques from AD rat hippocampus. To elucidate the therapeutic mechanisms of lavender, serum metabolic fingerprint of Aβ-induced rat Alzheimer’s models was investigated through nuclear magnetic resonance spectrometry.

**Methods:**

For the establishment of rat Alzheimer’s models, 10 *μg* of Amyloid beta 1–42 was injected to male Wistar rats. The lavender aqueous extract was injected 20 days after the establishment of the models, once daily for 20 days. Serum samples were collected and metabolite fingerprints were obtained using 500 *MHz* 1H-NMR spectrometry, following multivariate statistical analyses. The resulted metabolites were then subjected to pathway analysis tools to reveal metabolic pathways affected by the lavender extract treatment.

**Results:**

Levels of 10 metabolite markers including alanine, glutamine, serine, isoleucine, valine, carnitine, isobutyrate, pantothenate, glucose and asparagine were reversed nearly to control values after treatment with lavender extract. The results revealed that the most significantly affected pathways during treatment with lavender extract belonged to carbohydrate and amino acid metabolism, including pantothenate and CoA metabolism, glyoxilate and dicarboxylate metabolism, alanine, aspartate and glutamate metabolism, cysteine and methionine metabolism.

**Conclusion:**

As lavender extract reversed the direction of changes of some metabolites involved in AD pathogenesis, it was concluded that the extract might play a role in the disease improvement and serve as a potential therapeutic option for the treatment of AD. Moreover, the metabolites which were found in AD rats could serve as a potential marker panel for the disease; however, much further investigation and validation of the results is needed.

## Introduction

Alzheimer’s Disease (AD), a multifactorial neurodegenerative disorder, is the most prevalent cause of progressive loss of memory and cognitive impairments in elderly people^1–3^. Various environmental and genetic factors are involved in AD pathogenesis, one of the most important ones is oxidative stress^4^. Accurate and reliable diagnosis of AD can just be done by postmortem examination of brain tissue samples based on the presence of Amyloid beta (Aβ) and neurofibrillary tangles and hyper-phosphorylation of tau protein^5^. Currently, the most reliable body fluid for AD screening is the Cerebrospinal Fluid (CSF), which has its own challenges, especially in gathering method, lumbar puncture, which is still considered a relatively invasive collecting method and causes patient’s discomfort^6^. It is estimated that about 500 *ml* of CSF is secreted into blood daily, making blood a suitable source of biomarkers for neurodegenerative diseases like AD^7^. Thus, efforts are in progress to find novel valid markers in blood plasma or serum samples. Moreover, early detection of AD, is still a great challenge because the disease starts about 20 years or more before the emergence of the clinical symptoms. There are still no effective approaches for the treatment of AD. The most currently used drugs include Donepezil, Galantamine, Rivastigmine, and Memantine. These drugs act as acetylcholine esterase inhibitors, which improve the ability of the impaired nerves to transmit messages or as NMDA (N-methyl-D-aspartate) receptor antagonists, which regulate the activity of glutamate as an important neurotransmitter in learning and memory^8,9^.

In the past decades, herbal compounds have proven to be effective treatments for different diseases including AD^4,10–12^. The effect of these compounds on slowing the disease progression can be elucidated through metabolomic approaches which reveal the alterations in small molecule metabolites (<1500 *Da*) in the system under study^13^. These herbal medicines have antioxidant and anti-inflammatory properties as can be seen in compounds such as *Ginko biloba*^14,15^. Metabolic pathways are conserved through evolution and they have the highest levels of similarity between humans and rodents.

Pathway analysis of metabolomics data can propose mechanisms of actions of the herbal treatments. *Lavandula angustifolia* (lavender), a species from Lamiaceae family, is a plant with small purplish flowers which are mostly used to produce aromatic extracts^16^. Previous phytochemical studies demonstrated that the most effective components of the aerial parts of lavender are monoterpenes linalool and linalyl acetate, coumarins and triterpenoids which cause its pharmacoactivity^17–19^. It also contains aqueous phenolic compounds, such as hydroxycinnamic acids and flavone glycosides which are associated with its antioxidant activity^20^. Various pharmacological properties are attributed to lavender extract including anticonvulsant, sedative, analgesic, antioxidant and local anesthetic activity^21–24^.

In a recent investigation, reduction of L-dopa induced oxidative toxicity in mouse models was observed after treatment with *Lavandula angustifolia*^25^. Its aqueous extract has protective effects against glutamine-induced neurotoxicity in cell culture^19^. It was shown that lavender extract was effective in alleviating agitated behaviors in Chinese patients with dementia as an adjunctive therapy^22^. Several observations suggested that lavender extract may exert its physiological effects through disturbing dopaminergic neurotransmission^26^. Dopamine has important roles in brain functions, including locomotor activity, cognition, and emotion. Two recent studies revealed that lavender oil inhalation can improve scopolamine-induced memory impairment and dementia in the laboratory, male Wistar rats^27,28^. A recent study has investigated the effects of lavender floral water on the regulation of autonomeous nervous system and reducing stress^29^. Recently, Lavender extract has been shown to have the potential in clearing Aβ from AD rat hippocampus^30–32^. In the current study, therapeutic effects of the lavender aqueous extract on rat Alzheimer’s models are investtigated. Serum samples were collected from animals and metabolic fingerprinting was done by NMR methods to search for affected metabolic pathways during the herbal therapy.

## Materials and Methods

### Preparation of lavender extract

The aerial parts of Lavandula Angustifolia including flowers were collected in August 2015. A voucher was deposited at the Medicinal Plants and Drugs Research Institute (MPDRI), Shahid Beheshti University of Medical Sciences, Tehran, Iran, with the herbarium number 1092. The taxonomic identity of the plant was further confirmed by the institute’s botanist. Aqueous extract of *Lavandula angustifolia* was achieved according to Soheili M *et al*^33^. Briefly, flowers were air dried and weighed and placed into glass bottles. 250 *gr* of the dried flowers were mixed with 1 *L* of boiling water and stirred for 4 *hr*. The supernatant was collected by filtering through Whatman 3 *mm* filter paper. The filtrate was concentrated by vaporization.

### Animals

A total of 40 adults, male Wistar rats weighing 250–300 *gr* were employed. This study was approved by the ethical committee of Shahid Beheshti University of Medical Sciences. Treatment of the animals was in accordance with the policy of guide to the care and use of laboratory animals (NIH). The rats were kept in temperature controlled rooms under a 12 *hr*/12 *hr* light/dark cycle with access to water and food during the experiment. Wistar rats were randomly divided into the following 3 groups: 1- Normal controls (n=20), 2- Amyloid beta injected, as the Alzheimer’s group (n= 10), and 3- Amyloid beta injected with a following treatment with an aqueous lavender extract (n=10). Animal models of Alzheimer’s disease were created according to the procedure of Zali H *et al*^34^. Briefly, the animals were anesthetized with a combination of 100 and 5 *mg/kg i.p*. of ketamine and xylazine. The fresh powder of amyloid beta 1–42 mixed with 200 microliter of distilled water and incubated at 37°*C* for 7 days was used to create a fibrillar form of the peptide. The fibrillar form of amyloid beta 1–42 (Sigma Aldrich, St. Louis, MO, USA) was injected intracerebroventricularly (*i.c.v*.) 20 days before extract administeration. The injection site (AP=Bregma, LR=1.5 *mm*, D= 4 *mm*) was determined according to the Stereotaxic Atlas. The control animals were treated with the same procedure, except that they received distilled water. Alzheimer’s rats were treated with aqueous extract of lavender 20 days after the establishment of Alzheimer’s models. The optimum dosage of the aqueous extract was 200 *mg* per *kg* according to our previous studies^33–35^. The extract was injected once each day for 20 days at a volume of 0.4 *ml/kg* body weight. Morris Water Maze (MWM) was used as the screening test. The results of the MWM test have been published previously^33^.

### Serum sample preparation

Blood samples were collected by cardiac puncture. They were then remained 20 *min* in the refrigerator and centrifuged at 3000 *rpm* for 10 *min* at 4°*C* to separate the serum. The serum samples were kept in −80°*C* freezer until analysis.

### Instrumentation

The 1H-NMR analysis was the method of choice, on a Bruker DRX500 *MHz* spectrometer, equipped with 5 *mm* triple resonance inverse detection probe. 200 *μl* of D2O (99.9%, Sigma-Aldrich), was added to 300 *μl* of serum samples for the analysis. The chemical shifts of the samples were referenced to DSS (3-(Trimethylsilyl)-1-propanesulfonic acid-d6 sodium salt, 98% D, Sigma-Aldrich), as the standard, with peaks at 3.1 *ppm* (triplet), 1.9 *ppm* (pentet), and 0.8 *ppm* (triplet) at an intensity of 22% of the reference peak at 0.0 *ppm*. To reduce the broad signals of high molecular weight compounds, Carr-Parcell-Meibomian-Gill (CPMG) spin echo pulse with spin echo sequence π/2-tD-π-tD was carried out. Acquisition parameters included spectral width 8,389.26 *Hz*, relaxation delay 2 *s*, time domain points 32 *k*, scan number of 154, spectrum size 32 *k*, and acquisition time of 1.95 *s*. An exponential line broadening function of 0.3 *Hz* was applied to free induction decay prior to Fourier transformation. All the spectra were manually phase- and baseline-corrected using XWINNMR software (Bruker Spectrospin Ltd.).

### Data analysis

For the processing of the NMR spectra, ProMetab software (version 3_3), was used which works under the MATLAB environment (version 8.1.0.604, Mathworks, Cambridge, UK). The spectrum was divided into equal regions with the bucket size of 0.01 *ppm*, which yielded 813 spectral integral regions between 0.2 to 10 *ppm*. Signals related to presaturation of water resonance in the range of 4.5 to 5.5 were also removed. The spectra were normalized to reduce variations in sample concentrations. Multivariate statistical analyses, including Principal Component Analysis (PCA) and Partial Least Square-Discriminant Analysis (PLS-DA), were used to find significantly altered metabolites and to introduce a predictive model. The data processing and modeling were executed by the “Tool for statistical analysis on Microsoft Excel” which is a macros written by visual basic and is freely available at http://prime.psc.riken.jp/ ^36^. The 3D PCA and PLS-DA diagrams were plotted in R. The ROC curve is plotted using SPSS 16.0.

### Metabolite identification

The spectral bins of the highest importance according to Variable Importance in Projection (VIP) values were selected and the p-value and fold-change were calculated for each. Those bins with VIP values of more than 1.4, the p-value<0.05 and the fold change of more than 1.4 were brought to Biological Magnetic Resonance Data Bank (BMRB)^37^ for identification. Significantly altered metabolites were found with the matching of the selected NMR spectral bins with the available data in metabolomics database BMRB, with an accuracy of ±0.05 *ppm*.

### Metabolic pathway analysis

Pathway analysis was performed to introduce affected metabolic pathways in Alzheimer and lavender treated groups using the web server, Metaboanalyst (http://www.metaboanalyst.ca/)^38–40^. Pathway analysis was done by searching the database for Rattus norvegicus by hypergeometric overrepresentation test and topological analysis with the relative betweenness centrality method. The most significant pathways were selected according to their p-values and impact values calculated from topology analyses.

## Results

### Multivariate statistical analysis

As mentioned, processing of the NMR spectra was done by the ProMetab software. The result was a matrix including normalized spectral bins corresponding to different metabolites and the observations. The data were log10 transformed, and scaled by pareto method. Multivariate data analysis was performed on data matrix to find metabolites that mostly discriminated the groups, as potential biomarkers. A RIKEN platform in Microsoft Excel was used for the multi-variate analysis. Principal Component Analysis (PCA) was used as an unsupervised multivariate method. PCA is used for dimension reduction of data through making a linear combination of variables known as principal components and shows data structure as score and loadings plot. PCA analysis could reveal trends and groups of observations and find outliers. The variation which was explained by the first three components in PCA plots were 54.75, 11.87 and 8.7% for discriminating Alzheimer and control groups and 40.12, 23.57 and 9.7% for discriminating lavender-treated and Alzheimer groups. It was obviously observed in PCA plots that the two biological replicates in each comparison were well clustered together.

**Figure 1.**
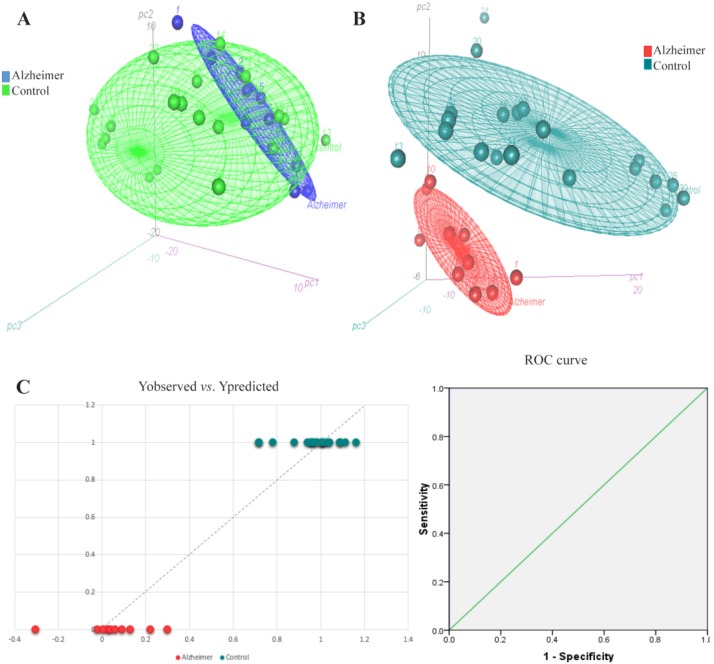
3D scatter scores plots for A) PCA and B) PLS-DA models for the comparison of Alzheimer rats versus control group. As can be seen, PLS-DA model could obviously discriminate Alzheimer and control groups. Part C presents the Y observed versus Y predicted and the resulting ROC curve with AUC of 1 after 7-fold cross-validation of the PLS-DA model which confirms the model has well separated the groups.

**Figure 2.**
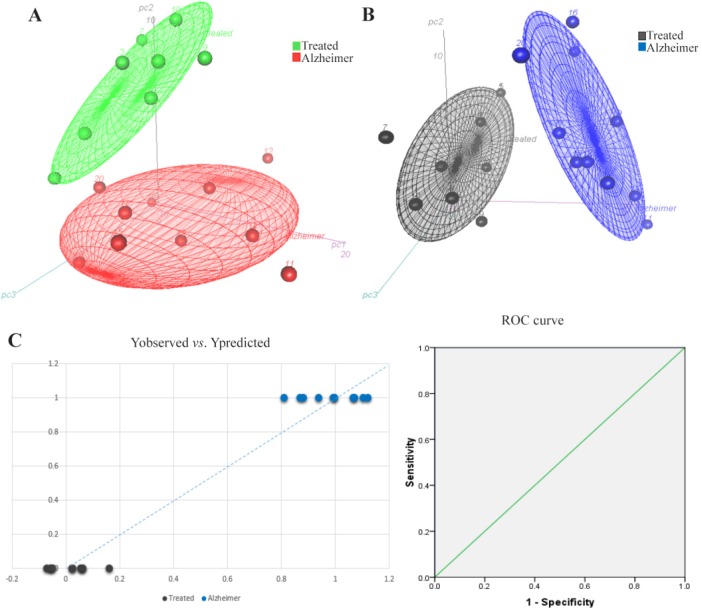
3D scatter scores plots for A) PCA and B) PLS-DA models for the comparison of lavender treated rats versus Alzheimer group. Part C, shows the Yobserved versus Ypredicted and the resulting ROC curve with AUC of 1 after 7-fold cross-validation of the PLS-DA model. The groups are well separated by the model.

Partial Least Square-Discriminant Analysis (PLS-DA), was the supervised regression method to find potential metabolite markers in Alzheimer and treated groups. PLS can find the variables which cause significant variations between clusters. It also demonstrates the goodness of separation between classes. A seven-fold cross-validation was used to calculate Q2 value for the model. Q2 displays the cumulative percent of variation in Y-matrix which was predicted by the model based on 7-fold cross-validation. The cumulative Q2 values of PLS-DA model was 0.803 in Alzheimer *vs*. control and 0.896 in treated *vs*. Alzheimer groups which demonstrated the model’s good predictive ability. The Root Mean Square Error (RMSE) values for these comparisons were 0.1455 and 0.0993, respectively. Figures 1 and 2 present the 3D scatter scoreplots for PCA and PLS-DA, since the model was constructed based on the three first components. Figures 1 and 2 part C, show Y “observed” versus Y “predicted” and the resulting Receiver Operating Characteristic (ROC) curve for the PLS-DA models. The Area Under the Curve (AUC) was 1 for both groups which shows the high accuracy of the model for the separation of classes.

### Selection of biomarkers

The VIP values extracted from the PLS-DA models were used to find the most significantly altered meta-bolites. VIP ranks the variables based on their contribution to the model. Metabolites with VIP values of more than 1.4, p<0.05 and fold-change of more than 1.4 were selected. Discriminating metabolite markers are presented in table 1. As can be seen, the most important metabolite markers for the Alzheimer group included alanine, glutamine, serine, valine, isobutyrate, carnitine, taurine, pantothenate, glucose, asparagine, succinate, glycine, creatinine, isoleucine, pyruvate, betaine, choline, scyllo-inositol and myo-inositol. Markers of lavender extract administration included alanine, glutamine, serine, valine, isobutyrate, glucose, carnitine, isoleucine, pantothenate, asparagine, trimethylamine, glycolate, methionine, citrate and acetate.

**Table 1.** Metabolite markers for the Alzheimer and lavender treated rats extracted from PLS-DA model. Those metabolites which had VIP values>1.4, p-value<0.05 and fold-change>1.4 have been considered. (*p<0.05, **p<0.01, ***p<0.001)

	**Metabolite name**	**BMRB ID**	**Alzheimer *vs*. control**			**Treated *vs*. alzheimer**		

			**VIP value**	**Fold change**	**p-value**	**VIP value**	**Fold change**	**p-value**
**1**	Alanine	bmse000282	1.622	9	0.0134^*^	1.922	0.164	0.00005^***^
**2**	Glutamine	bmse000038	1.470	2.6	0.0008^***^	1.605	0.4	0.0094^**^
**3**	Serine	bmse000048	1.910	0.47	0.0011^**^	1.724	1.42	0.0002^***^
**4**	Valine	bmse000052	2.750	3.88	0.0004^***^	2.601	0.48	0.0019^**^
**5**	isobutyrate	bmse000439	1.661	0.6	0.0095^**^	1.423	1.4	0.0025^**^
**6**	Carnitine	bmse000211	1.789	3.1	0.0005^***^	1.558	0.43	0.0035^**^
**7**	Pantothenate	bmse000287	1.910	0.47	0.0011^**^	1.724	1.4	0.0002^***^
**8**	Glucose	bmse000015	2.132	2	0.0001^***^	2.763	0.4	0.00003^***^
**9**	Asparagine	bmse000030	1.900	0.45	0.001^**^	1.526	3.48	0.0081^**^
**10**	Isoleucine	bmse000041	1.474	0.6	0.0217^*^	1.424	1.44	0.0025^**^
**11**	succinate	bmse000183	1.661	0.62	0.0095^**^	-	-	-
**12**	Glycine	bmse000089	1.427	1.4	0.0011^**^	-	-	-
**13**	Creatinine	bmse000155	1.472	0.6	0.0068^**^	-	-	-
**14**	Pyruvate	bmse000112	2.060	0.47	0.0002^***^	-	-	-
**15**	Betaine	bmse000069	1.699	2.6	0.0042^**^	-	-	-
**16**	Choline	bmse000285	1.472	0.6	0.0068^**^	-	-	-
**17**	Scyllo-inositol	bmse000113	1.699	2.57	0.0042^**^	-	-	-
**18**	Myo-inositol	bmse000103	1.427	1.4	0.0011^**^	-	-	-
**19**	Taurine	bmse000120	1.655	1.64	0.0002^***^	-	-	-
**20**	Trimethylamine	bmse000224	-	-	-	1.603	2.44	0.0038^**^
**21**	Glycolate	bmse000245	-	-	-	1.806	1.6	0.00002^***^
**22**	Methionine	bmse000044	-	-	-	2.172	1.7	0.0002^***^
**23**	Citrate	bmse000076	-	-	-	1.958	6.5	0.0005^***^
**24**	Acetate	bmse000191	-	-	-	2.596	0.48	0.0004^***^

### Metabolic pathway analysis

To elucidate the Alzheimer’s related pathways and also mechanisms of action of the lavender extract on the rat models of AD, biochemical pathways were investigated using Metaboanalyst-3 server^38–40^ which combines the results of pathway enrichment analysis and pathway topology to find the most relevant metabolic pathways. The most important pathways in Alzheimer’s disease included glycine, serine and threonine metabolism, alanine, aspartate and glutamate metabolism, aminoacyl-tRNA biosynthesis, methane metabolism, valine, leucine and isoleucine metabolism, pantothenate and CoA metabolism, TCA cycle, and cysteine and methionine metabolism. The most significant pathways related to lavender administration in rats also included aminoacyl-tRNA biosynthesis, alanine, aspartate and glutamate metabolism, pantothenate and CoA metabolism, glyoxilate and dicarboxylate metabolism, cysteine and methionine metabolism. The detailed results are shown in table 2.

**Table 2.** The result of pathway analysis using Metaboanalyst3, for Alzheimer’s disease and also lavender-treated groups. The pathways with p-value<0.05 and impact value of more than 0 were selected.(*p<0.05, **p<0.01, ***p<0.001)

**Metabolic pathway category**	**Pathway name**	**Metabolite matches**	**p-value**		**Impact**	

			**AD**	**Treated**	**AD**	**Treated**
**Carbohydrate metabolism**						
	pantothenate and CoA metabolism	Pantothenate, Valine	0.011*	0.009**	0.02	0.02
	TCA cycle	Succinate, Pyruvate	0.02*	-	0.097	-
	glyoxilate and dicarboxylate metabolism	Glycolate, Citrate	-	0.01*	-	0.333
**Amino acid metabolism**						
	glycine, serine and threonine metabolism,	Serine, Choline, Glycine, Betaine, Pyruvate	1.64E-5***	-	0.534	-
	alanine, aspartate and glutamate metabolism	Alanine, Glutamine, Pyruvate, Succinate, Asparagine	1.05E-4***	0.0014**	0.149	0.149
	Valine, leucine and isoleucine metabolism	Valine, Pyruvate, Isoleucine	0.006**	-	0.333	-
	cysteine and methionine metabolism	Serine, Pyruvate, Methionine	0.038*	0.03*	0.044	0.117
**Genetic information processing**						
	aminoacyl-tRNA biosynthesis	Glutamine, Glycine, Valine, Serine, Alanine, Asparagine, Methionine	6.22E-4***	2.11E-5***	0.137	0.137
**Energy metabolism**	Methane metabolism	Glycine, Serine	0.004**	-	0.4	-

## Discussion

Rat models of AD are applicable in the study and modeling of the mechanisms responsible for the pathogenesis of the human disease^3^. Various drugs are currently used for the treatment of AD. In recent years, attentions are drawn to the use of herbal medicine in the treatment of cognitive impairments^11,12^. The Lavender extract has been shown to have therapeutic effects such as anticonvulsant, sedative, analgesic, anti-oxidant and local anesthetic activity^18^. It has been recently revealed that Lavender extract has the ability of clearing Aβ plaques from AD rat hippocampus^30^. Thus, the effects of aqueous extracts of lavender on the improvement of memory and cognition were examined in Aβ-induced rat Alzheimer’s models through metabolomics investigations of the serum, utilizing Nuclear Magnetic Resonance (NMR) spectroscopy. NMR has the advantage of high repeatability and the wide range of identifying metabolites. Moreover, significant metabolites which were discovered in AD models compared to healthy controls could serve as a potential marker panel for detection of AD.

Serum metabolite profiles of 3 groups of rats (control, AD, and AD treated with lavender extract) were compared and significantly altered metabolites between the groups were identified which are presented in table 1. The extract-treated AD rats showed memory and cognitive abilities improvements revealed by Morris-Water-Maze test. The MWM test results have been published elsewhere^33^. The treated group had also reversed direction of changes in some of metabolites levels, including alanine, glutamine, serine, glucose, asparagine, isoleucine, valine, carnitine, isobutyrate and pantothenate compared to AD group which showed the effectiveness of the lavender extract on the improvement of AD rats.

Seeking for the mechanisms of action of the herbal extract, biochemical pathways corresponding to altered metabolites were found using the Metaboanalyst online server. This platform uses KEGG metabolic pathways as the knowledge-based, integrating univariate and over-representation analyses and also novel algorithms such as global test, global Ancova and network topology in the pathway analysis. The altered metabolic pathways mostly belonged to amino acids and carbohydrate metabolism, including alanine, aspartate and glutamate metabolism, pantothenate and CoA metabolism, glyoxilate and dicarboxylate metabolism, cysteine and methionine metabolism. The main alterations in AD pathogenesis are related to energy metabolism, through different pathways such as TCA cycle, glycollysis, and amino acid metabolism, and also dysfunction in mitochondrial activities. Impairments in energy metabolism were indicated by alterations in serum levels of glucose, glycine, serine, asparagine, succinate and isobutyrate. Impairments of TCA cycle enzymes and decreased levels of pyruvate dehydrogenase complex, α-ketoglutarate dehydrogenase complex, isocitrate dehydrogenase, and malate dehydrogenase had been reported in AD^41^. Alterations in TCA cycle intermediate levels have also been previously shown in AD^42^.

A previous study demonstrated that high serum glucose levels are associated with cerebral hypometabolism in brain AD regions^43^. Another study described shared mechanisms between type 2 diabetes and AD, especially in glucose homestasis^44^. In our study, asparagine level has decreased in AD group which is along with previous studies which indicates energy imbalance has occurred in the disease. The decrease of TCA cycle metabolites such as succinate is also because of mitochondrial impairments which lead to energy deficiency^45,46^. Changes in acetate concentration has also occurred in AD pathogenesis. Ketone bodies such as acetoacetate, β-hydroxybutyrate, and their related compound, acetate, are used to produce energy *via* TCA cycle and conversion to acetyl coenzyme A. It has been previously shown that AD mouse models consume ketone bodies to produce ATP during mitochondrial-related energy deficiency by the increased activity of oxoacid-CoA transferase 1 enzyme, which leads to decreased β-hydroxybutyrate levels as a ketone body and increased levels of by-products of ketone body metabolism^47^.

One of the pathways related to carbohydrate metabolism is the inositol phosphate metabolism. Inositol phosphates such as myo-inositol and scyllo-inositol involve in various cellular functions including signaling processes, cell growth and differentiation, endo-cytosis, and apoptosis. Altered levels of myo-inositol are related to alterations in phosphatidyl inositol metabolism and dysfunction of the phosphoinositide signaling system. Increased activity of myo-inositol mono-phosphate was previously reported in AD patients’ brains^48^. Altered homeostasis of myo-inositol in neurons leads to the accumulation of free myo-inositol in the serum^49^.

One of the altered metabolites in AD group was taurine. Taurine (2-aminoethane sulfonic acid), is a non-protein amino acid implicated in various biological functions in the human body which is non-essential in rodents but essential in humans. It is the most abundant free amino acid in the intracellular compartments of human and other animals and is also an end-product in the cysteine metabolism. It is isolated in small amounts from brain synaptosomes in the form of glutaurine, which acts as a neurotransmitter. It also stabilizes the plasma membrane and affects glutamate generation which is also a very important neurotransmitter. Taurine has physiological functions such as anti-inflammatory, osmolarity maintenance, antioxidant and detoxification activities which are associated with Alzheimer’s pathogenesis^50,51^. Acetylcholine is a neurotransmitter with low amounts in AD patients. Taurine has been shown to be able to increase the acetylcholine level in the brain. Various researchers have shown the decreased amounts of taurine in advanced AD patients^52^.

Amino acid metabolism pathways were also altered during treatment of AD rat models including alanine, aspartate and glutamate metabolism and cysteine and methionine metabolism. Cystine is the major form of cysteine in physiological conditions, and a limiting factor in the glutathione biosynthesis. Reduced glutathione is an antioxidant in the brain, which protects the brain cells against oxidative stress and excitotoxicity of glutamate^53^. Alanine is the downstream of N-acetyl-aspartate which is an important neurotransmitter. It is commonly produced by amination of pyruvate by the enzyme alanine transaminase, which involves interconversion of alanine and pyruvate, and α-ketoglutarate and glutamate. Alanine participates in cellular bioenergetics, lower levels of which in AD group demonstrates the energetic deficiency and hypometabolism in AD, which could be related to decreased carbohydrate metabolism and mitochondrial dysfunction^54^. Aspartate is also synthesized by the amination of oxaloacetate. It is converted to the asparagine by the enzyme asparagine synthase. Aspartate is involved in cellular signaling processes in different forms, including beta-alanine, adenylo-succinate, and arginino-succinate. Aspartate also acts as a brain neurotransmitter which activates NMDA receptors. Glutamate is a precursor for the synthesis of neurotransmitter GABA. It is also the most abundant excitatory neurotransmitter in the human CNS. After releasing from synaptic vesicles, it will bind NMDA receptors for activating the cells. Altered glutamine level is related to neurotransmitter system^55^. Glycine, serine and threonine metabolism, also alters during AD pathogenesis. This pathway participates in the synthesis and breakdown of glycine, serine, threonine and cysteine. Glycine and cysteine are synthesized from serine. Glycine is an inhibitory neurotransmitter in the nervous system. In our study, altered levels of glycine, serine, betaine, choline and pyruvate were observed, which are related to this metabolic pathway. It has been recently demonstrated that betaine rescues neuron damage. In previous studies, it was shown that betaine attenuated Alzheimer pathological changes and memory deficits induced by homo-cysteine in AD rat models^56^. In another study, it was revealed that betaine could reduce Aβ levels in the brain by altering amyloid precursor protein processing in N2a cell line and proposed that it could have a protective role in amyloid beta production^57^. L-serine levels were also decreased in our study. Previous investigations have studied the role of D-serine in the pathogenesis of AD^58–60^. They have shown that D-serine levels were elevated in hippocampus and parietal cortex in AD rat models. D-serine is produced from L-serine by the enzyme serine racemase which can be a cause of the decreased serum levels of L-serine as was observed in our study. Pantothenate and CoA metabolism was the other important metabolic pathway. Pantothenic acid is essential for the synthesis of neurotransmitter acetylcholine, decreased levels of which in our study demonstrated its role in AD pathogenesis. In a study of J. Cooper^61^, it was observed that altered levels of pantothenate occurred in a cohort of patients with dementia and proposed that it could be related to dysregulation of the Sodium-Dependent Multivitamin Transporter (SMVT) across cell membranes in AD.

## Conclusion

**Figure 3.**
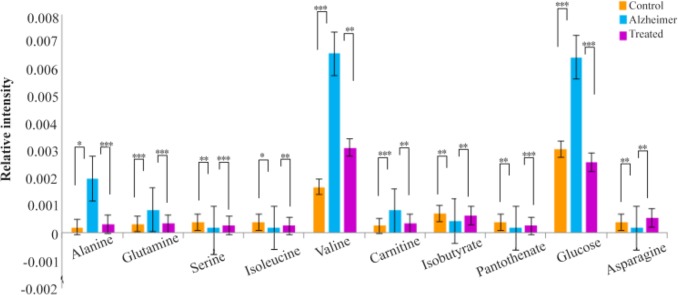
10 biomarkers levels were reversed nearly to control values after treatment with lavender extract. The Y-axis represents the relativepeak intensities in NMR spectra (*p<0.05, **p<0.01, ***p<0.001).

To the best of our knowledge, this is the first study that investigated the therapeutic effects of lavender extract on the memory improvement in Aβ-induced rat models of Alzheimer’s disease through NMR metabolic fingerprinting of the serum. As was observed by Morris-Water-Maze test, the aqueous lavender extract could successfully improve memory and cognitive abilities in AD rats. It was also observed that values of some metabolites in lavender-treated group, including alanine, glutamine, serine, isoleucine, valine, carnitine, isobutyrate, pantothenate, glucose and asparagine reversed to control values which proposed the effectiveness of the therapy (Figure 3). The mechanism of action of the treatment was elucidated through the identification of affected metabolic pathways using significantly altered metabolites between AD and lavender-treated AD groups. The most significantly affected pathways belonged to carbohydrate and amino acid metabolism, including alanine, aspartate and glutamate metabolism, pantothenate and CoA metabolism, glyoxilate and dicarboxylate metabolism, and cysteine and methionine metabolism, so it could be concluded that the lavender extract exerts its therapeutic effect by balancing energy metabolism and also the levels of neurotransmitters.

According to the study results, lavender extract could be introduced as an effective drug candidate for the treatment of AD. According to the need for biomarkers of AD specially in blood samples which has a less invasive method than collecting CSF fluid, also a serum metabolite marker panel was prpoposed for the detection of AD, including alanine, glutamine, serine, valine, isobutyrate, carnitine, taurine, pantothenate, glucose, asparagine, succinate, glycine, creatinine, isoleucine, pyruvate, betaine, choline, scyllo-inositol and myo-inositol. However, these metabolites are required to be validated and studied in large cohorts.
